# Two-pore Channels Enter the Atomic Era: Structure of Plant TPC Revealed

**DOI:** 10.1016/j.tibs.2016.04.007

**Published:** 2016-06

**Authors:** Sandip Patel, Christopher J. Penny, Taufiq Rahman

**Affiliations:** 1 Department of Cell and Developmental Biology, University College London, London, UK; 2 Department of Pharmacology, University of Cambridge, Cambridge, UK

## Abstract

Two-pore channels (TPCs) are intracellular Ca^2+^-permeable ion channels that are expressed on acidic Ca^2+^ stores. They are co-regulated by voltage and Ca^2+^ in plant vacuoles and by the second messenger NAADP in animal endo-lysosomes. Two new studies of plant TPC structures reveal essential features of their architecture and provide mechanistic insight into their workings.

TPCs are key members of the voltage-gated ion channel superfamily 1, 2. By virtue of their tandem domain architecture, they likely represent the long-hypothesised evolutionary intermediate between the extensively studied one- and four-domain channels exemplified by voltage-gated K^+^ and Ca^2+^/Na^+^ channels, respectively (Figure 1A) [3]. In plants, TPCs localise to the vacuole and mediate slow-vacuolar currents, which regulate processes including stomatal movement, germination and salt stress-induced Ca^2+^ waves [2]. In animals, they localise to the analogous endo-lysosomal system and are activated by the Ca^2+^ mobilising messenger NAADP in addition to the phosphoinositide PI(3,5)P_2_ to control numerous Ca^2+^-dependent outputs [1]. Here, we spotlight two recent studies that report congruent crystal structures of TPC1 from *Arabidopsis thaliana* at 3.3 Å [4] and 2.87 Å [5] resolution, providing the first atomic insight into these channels.

Plant TPCs are non-selective ion channels [2]. The new structures reveal selectivity filters that lack glutamate and aspartate residues 4, 5. In the ion-selective voltage-gated Ca^2+^ and Na^+^ channels, these residues use their negatively-charged sidechains to coordinate permeating ions [3]. Thus, their absence in TPC perhaps explains the lack of ion selectivity. Indeed, ions are not observed within the selectivity filter itself. Instead, ions are found on either side of the selectivity filter, coordinated within the vestibule between the selectivity filter and the ‘bundle crossing’ that likely mediates channel opening, and also at the luminal mouth of the channel. Asparagine side chains within the selectivity filter of domain II project towards the ion permeation pathway, similar to that in a structural homology model of animal TPCs [3]. This creates the narrowest constriction within the pore (∼4.7 Å), wide enough for hydrated Na^+^ and Ca^2+^ ions to pass through. Interestingly, a glutamate residue previously shown to alter ion selectivity of mouse TPC2 [6] appears to project away from the permeation pathway.

Plant TPCs are voltage-gated in a Ca^2+^-dependent manner [2]. Cytosolic Ca^2+^ binds to the second of the two EF hands in the cytosolic linker connecting the two domains, promoting channel opening by reducing voltage activation towards hyper-polarising potentials. In the new structures, the first helix of EF hand 1 is contiguous with the extended S6 helix of domain I, providing a means of conveying Ca^2+^-dependent conformational changes in the EF hands to the pore region (Figure 1B). However, Ca^2+^ binding to EF hand 1 is likely structural, with cytosolic Ca^2+^ binding to EF hand 2 (resolved in [5]) mediating channel activation. Intriguingly, EF hand 2 connects to the distal C terminus, which also contributes to Ca^2+^ coordination, and the C terminus of the adjacent subunit via salt bridges (Figure 1B). A region of the N terminus harbouring a phosphorylation site immediately prior to S1 of domain I additionally interacts with the EF hands [5] (Figure 1B). Such intricate intramolecular interactions may explain lack of activity of N-terminally tagged TPC1 in animals [7].

Despite the presence of two voltage sensors, only the second one appears to be functionally relevant. Thus, mutation of the positively-charged residues in S4 of domain II, which classically mediate voltage gating, reduces voltage sensitivity, unlike equivalent domain I mutations [4]. Additionally, anionic residues in S2 and S3, which serve as counter ions for the positively charged residues in S4, are absent in domain I. Of note, the structures offer the first view of a voltage sensor in a resting state, which is of major relevance to understanding voltage gating within the voltage-gated ion channel superfamily. Interestingly, the first gating charge and two of the four counter ions in domain II [4] are not conserved in human TPC1, despite reported voltage-gating [8]. Indeed, arginine residues in human TPC1 subjected to site-directed mutagenesis [8] align to the S4-S5 linker in domain I of plant TPC, which forms a salt bridge with the N terminus [5]. These data suggest that the reported loss-of-function in mutant human TPC1 may be indirect. The emerging picture of plant TPC1 gating is of channel opening achieved by the coordinated movement of S6 in domains I and II through the actions of Ca^2+^ and voltage, respectively.

In contrast to the activating effects of cytosolic Ca^2+^, luminal Ca^2+^ inhibits plant TPC opening [2]. The crystal structures reveal three luminal Ca^2+^ binding sites in the voltage sensors 4, 5 (Figure 1C), which are mostly conserved in human TPCs. One of these sites is in the inactive voltage sensor in domain I and its functional relevance remains to be established [5]. The other two sites are in the active voltage sensor in domain II, in proximity to the aspartate residue that is substituted by arginine in the well-described *Fou2* mutation (Figure 1C). This mutation abolishes Ca^2+^-dependent inhibition [2]. The key Ca^2+^ binding site clamps the otherwise dynamic voltage-sensing S4 helix to the S1/S2 loop, thereby providing an elegant mechanism for luminal Ca^2+^ inhibition of plant TPCs [4].

Intriguingly, the structure reported in [5] is in complex with Ned-19, an NAADP antagonist identified by shape and electrostatic similarity [9]. Ned-19 is proposed to clamp the pore regions of one subunit to the active voltage sensor domain in domain II of the other subunit. However, unlike luminal Ca^2+^, Ned-19 does not interact with the voltage-sensing S4 but rather with S1 of domain II (Figure 1C), which remains static during voltage gating. Physiologically, NAADP neither regulates plant TPCs [10] nor binds directly to animal TPCs [1]. It is therefore unclear whether NAADP would interact at the Ned-19 binding site in plant TPCs. In the absence of functional data however, it is worth noting that Ned-19 antagonises NAADP action in a non-competitive manner [9] suggesting the presence of additional binding sites, possibly within a subunit interface as proposed [5]. The structure reported in [5] also reveals a fatty acid (modelled as palmitic acid) adjacent to the site for luminal Ca^2+^ inhibition (Figure 1C). Interestingly, plant TPC1 is inhibited by polyunsaturated fatty acids [11] and an early report suggests inhibition of NAADP-evoked Ca^2+^ release by arachidonic acid [12]. Whether such regulation equates to the identified site requires further experimental analyses.

The past few years have witnessed dramatic advances in the structural biology of voltage-gated ion channels. The work by the Jiang and Stroud groups provide key fundamental insights into TPCs from plants. The structure of animal TPCs is eagerly awaited, in particular due to their emergence as potential therapeutic targets [1].

## Figures and Tables

**Figure 1:**
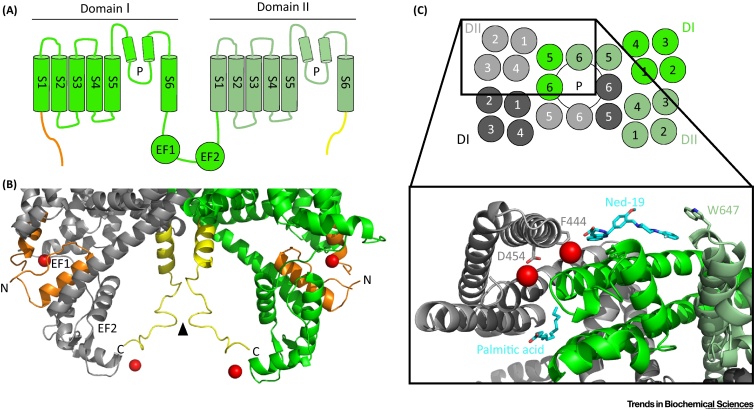
Structure of plant TPC. (A) Cartoon depicting an individual subunit of plant TPC1 comprising two repeated domains (DI and DII) of 6 transmembrane helices (S1-S6) and a re-entrant pore loop (P), connected by a cytosolic linker harbouring two EF-hands (EF1 and EF2). (B) Side view of TPC highlighting binding sites for Ca^2+^ (red spheres) within the two EF hands. The first and second EF hands are in proximity to a helix within the N terminus (orange) and the C terminus (yellow), respectively. Inter-subunit interaction between the C termini is marked by the arrowhead. (C) Cartoon depicting assembly of the TPC dimer (top). Inset is a luminal view of the TPC structure highlighting binding sites for Ca^2+^ (red spheres), Ned-19 and palmitic acid (both in cyan). The two TPC subunits are coloured grey and green. One of the three luminal Ca^2+^ ions is coordinated by D454 (mutated in *Fou2*) at the top of SI in DII. Ned-19 binds at an inter-subunit interface that includes residues from the voltage sensor of DII in one subunit (F444 in S1) and both pore domains of the adjacent subunit (*e.g.*, W647 in S6 of DII).
